# Identification of Arbuscular Mycorrhiza Fungi Responsive microRNAs and Their Regulatory Network in Maize

**DOI:** 10.3390/ijms19103201

**Published:** 2018-10-16

**Authors:** Yunjian Xu, Suwen Zhu, Fang Liu, Wei Wang, Xuewen Wang, Guomin Han, Beijiu Cheng

**Affiliations:** 1 School of Life Sciences, Anhui Agricultural University, Hefei 230036, China; xuyunjian1992@163.com (Y.X.); zhusuwen@126.com (S.Z.); wangweisys@ahau.edu.cn (W.W); 2 The National Engineering Laboratory of Crop Stress Resistance Breeding, Anhui Agricultural University, Hefei 230036, China; weishanren163@163.com; 3 Department of Genetics, University of Georgia, Athens, GA 30602, USA; xwwang@uga.edu

**Keywords:** arbuscular mycorrhiza symbiosis, miRNA, maize, deep sequencing analysis, regulatory network

## Abstract

Maize can form symbiotic relationships with arbuscular mycorrhiza (AM) fungus to increase productivity and resistance, but the miRNAs in maize responsible for this process have not been discovered. In this study, 155 known and 28 novel miRNAs were identified by performing high-throughput sequencing of sRNA in maize roots colonized by AM fungi. Similar to the profiles in other AM-capable plants, a large proportion of identified maize miRNAs were 24 nt in length. Fourteen and two miRNAs were significantly down- and up-regulated in response to AM fungus *Glomus intraradices* inoculation, respectively, suggesting potential roles of these miRNAs in AM symbiosis. Interestingly, 12 of 14 significantly down-regulated known maize miRNAs belong to the miR399 family, which was previously reported to be involved in the interaction between *Medicago truncatula* and AM fungi. This result indicated that the miR399 family should regulate AM symbiosis conservatively across different plant lineages. Pathway and network analyses showed that the differentially expressed miRNAs might regulate lipid metabolism and phosphate starvation response in maize during the symbiosis process via their target genes. Several members of the miR399 family and the miR397 family should be involved in controlling the fatty acid metabolism and promoting lipid delivering from plants to AM fungi. To the best of our knowledge, this is the first report on miRNAs mediating fatty acids from plant to AM fungi. This study provides insight into the regulatory roles of miRNAs in the symbiosis between plants and AM fungi.

## 1. Introduction

Plant microRNAs (miRNAs) are a class of short non-coding RNAs, generally 21–24 nt, that regulate the transcripts of target genes at transcriptional and post-transcriptional levels by forming the RISC (RNA-induced silencing complex) [1,2]. MiRNA precursors in plant are generally transcribed by RNA polymerase II and processed by DCL1 (DICER-LIKE 1) to generate mature miRNAs [3]. MiRNAs play important roles in plant development, nutrient signaling, hormonal signaling, organogenesis, and resistance to abiotic stresses [4,5]. For example, the expression of miR399 is strongly induced by phosphate (Pi) deprivation, and the overexpression of miR399 in Pi-deprived *Arabidopsis* leads to a high Pi accumulation in leaf [6]. Besides, several miRNAs have been reported to be involved in biotic stresses [7]. MiR393 in *Arabidopsis* was the earliest identified miRNA which could control pathogen resistance by negatively regulating transcripts of auxin receptors [8]. MiRNA863-3p in *Arabidopsis* positively regulates plant immunity upon infection by sequentially targeting negative regulators [9].

A few studies reported that miRNAs play vital roles in plant–microbe symbiosis. Plant and arbuscular mycorrhiza (AM) fungi symbiosis is one of the most investigated plant–microbe interaction [10]. The mutual relationships with AM fungi are widespread among land plants, which assist the uptake of nutrients from the soil [11]. However, the symbiosis process is tightly controlled by the plant to avoid over-colonization in the roots by AM fungi [12]. Several plant genes, e.g., *NSP1* (Nodulation Signaling Pathway 1), and *NSP2* involving in the establishment and maintenance of plant-microbe symbioses, have been identified [13,14]. During the plant–microbe interaction process, some miRNAs are important regulators of the symbiotic genes. For instance, miR171h from *Medicago truncatula* controls the colonization of AM fungi by down-regulating *NSP2* [15]. MiR171b is expressed exclusively in root cells containing arbuscules and stimulates AM symbiosis [12]. MiR396 from *M. trunctula* regulates root architecture and symbiosis with AM fungi by targeting *MtGRF* (Growth regulating factor) [16]. MiR399 is accumulated in the roots of plant *M. trunctula* and tobacco (*Nicotiana tabacum*) during AM symbiosis [17]. LOST MERISTEMS 1 (LOM1), negatively regulated by miRNA171 family members, is a positive regulator of AM symbiosis in the control of root colonization and arbuscule abundance [12]. However, miR171b has a mismatched target site only in species which form AM symbiosis, and protects LOM1 from cleavage by other miR171 family members and thereby enables AM symbiosis [12]. In addition, plant miRNAs involved in nodulation were also uncovered. MiR166 and miR169 can mediate nodulation in *M. trunctula* [1]. MiR172c of soybean and *Lotus japonicus* regulates the rhizobium infection and nodule organization [18].

Although AM-related miRNAs have been discovered mainly in legumes, investigation on the interaction in Poaceae plants was less performed. Maize is one of the important crops in the world. As a non-legume plant, maize is an excellent plant which is often used for propagation of AM fungal spores. Recently, several studies have surveyed miRNA composition in maize in response to Pi deficiency [19,20], low nitrate [21], drought [22], and salt [23]; however the AM symbiotic conditions were ignored in their investigations. As far as we know, there is no available report on characterization of miRNAs response to AM fungi in maize roots. In the present study, several AM fungi-responsive miRNAs in maize roots were identified via high-throughput sequencing. Furthermore, the symbiosis related miRNA-target genes and their metabolic pathways were also analyzed.

## 2. Results

### 2.1. Sequencing of Small RNAs in Maize Roots

**Figure 1:**
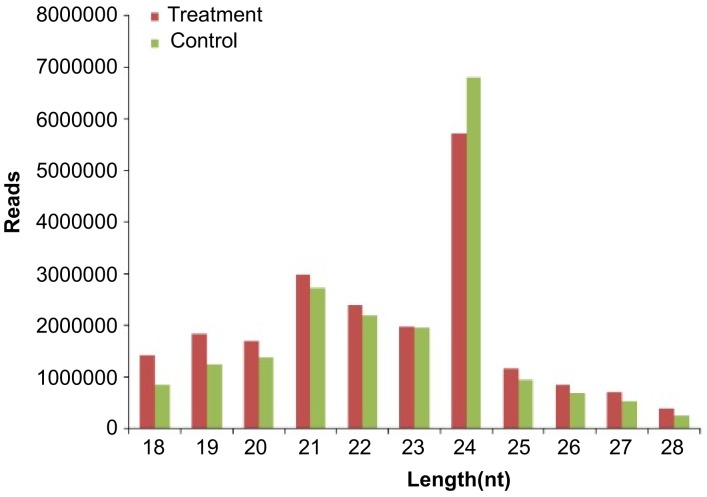
Read length distribution of small RNAs.

To study the role of miRNAs during the development of mycorrhiza, small RNA libraries derived from the maize roots with or without AM fungi inoculation were sequenced (Figure S1). Three biological replications were used for each treatment. After the removal of adaptor sequences and low-quality reads, 1,332,127 and 1,460,953 high-quality clean reads, on average, were obtained from fungus *Glomus intraradices* (also called *Rhizophagus irregularis*)—infected samples and the control samples, respectively (Table S1). 90.28% reads from fungal infected roots were perfectly mapped onto maize genome, while 83.67% reads from roots without fungal inoculation were perfectly mapped (Table S1). More than 72% small RNA sequences in the six libraries were 20–28 nt long. In particular, 69% and 76% of the sRNAs had a length between 20 and 24 nt in the treated maize root and control, respectively (Figure 1, Table S2). The most abundant sRNAs were 24 nt in length across all samples, followed by 21 and 22 nt (Figure 1, Table S2); 2043.5, 1692, 208.5, 4658.5 and 12529.5 unique reads, on average, in six libraries, were found to belong to rRNA, snoRNA, tRNA, snRNA and other non-coding RNAs, respectively (Table S3).

### 2.2. Identification of Known Maize miRNAs

**Figure 2:**
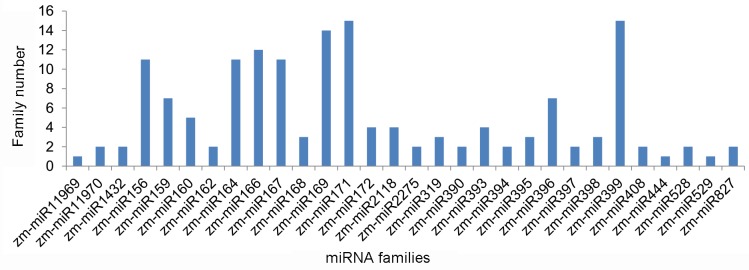
The number of family members and the abundance of miRNA families.

A total of 155 known maize miRNAs belonging to 30 miRNA families were identified (Table S4). The number of the family member was varied in the roots with or without inoculated *G. intraradices* to some extent (Figure 2). In the roots from 40 days after inoculation (DAI), 15 members were detected in two miRNA families (zm-miR171, zm-miR399), followed by the zm-miR169 family with 14 members. Two miRNA families, zm-miR444 and zm-miR529, have only one member.

### 2.3. Prediction of Novel miRNAs

After removing the known miRNAs in the sequencing data set, we conducted the *de novo* prediction using the remaining data set with ShortStack pipeline (Accessed online: http://sites.psu.edu/axtell/software/shortstack/). Twenty eight novel miRNAs were identified (Table S5, Supplementary sequence files). Five of the novel miRNAs can be classified into currently known miRNA families in the miRNA database while the remaining 23 miRNAs had no similarity to any known family. Therefore, we classified them as novel miRNAs. The length of the novel miRNA precursors ranges from 83 to 268 nt (Supplementary sequence files).

### 2.4. Differentially Expressed miRNA (DEM) Responsive to Arbuscular Mycorrhiza (AM) Fungus

**Figure 3:**
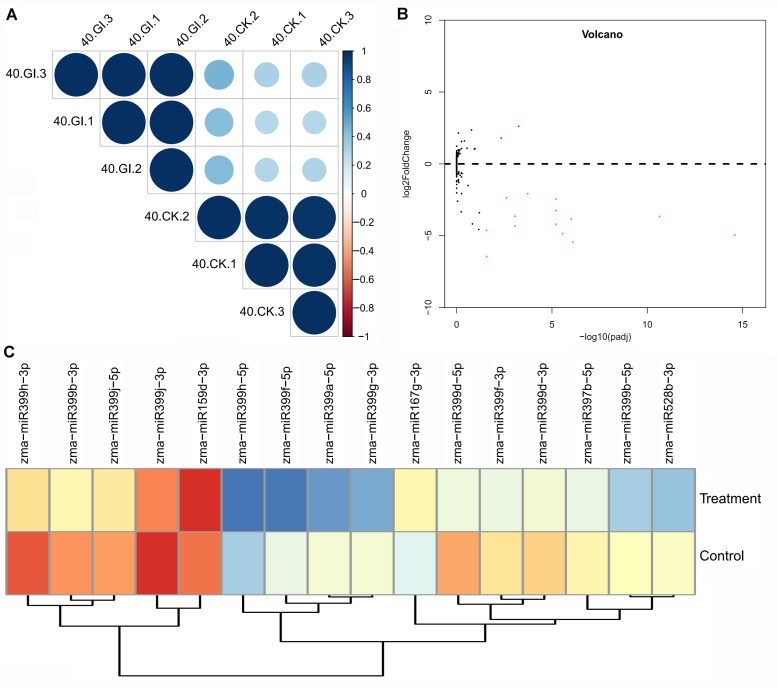
Correlation and expression difference among samples. (**A**) Correlation between different biological replicates. 40.GI, 40-day root with *G. intraradices* inoculation; 40.CK, 40-day root without *G. intraradices* inoculation. 1, 2, 3 represents replicate numbers. (**B**) Volcano plots displaying differentially expressed miRNAs between two samples. The significantly up- and down-regulated miRNAs are shown as the red dots and green dots, respectively. (**C**) Heatmap of the differentially expressed miRNAs. Treatment, roots with *G. intraradices* inoculation; Control, roots without *G. intraradices* inoculation. The color scale represents the relative expression level of differentially expressed miRNAs.

**Figure 4:**
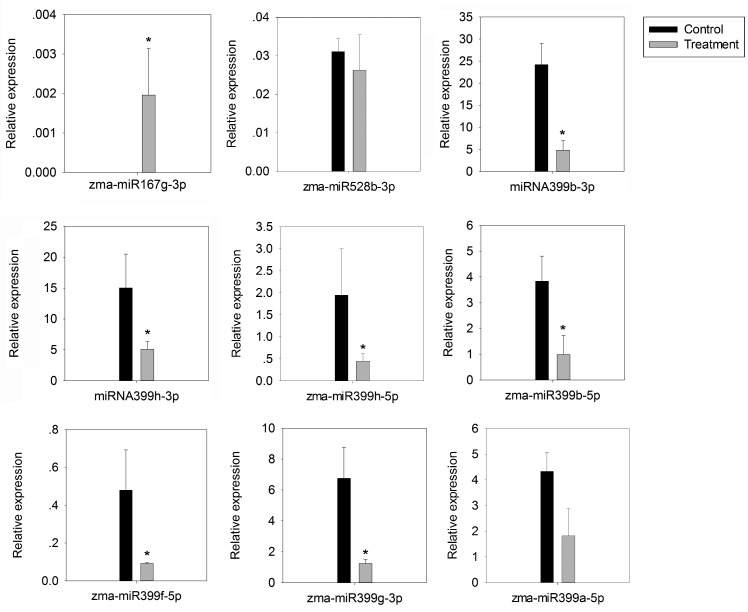
Quantitative reverse transcription polymerase chain reaction (qRT-PCR) validation of expression profiles of nine miRNAs. The house-keeping gene *5S rRNA* was used as the internal control, and Error bars represent SD from three replicates. Asterisk indicates statistically significant differences between control and AM infected maize (* *p* < 0.05). Student *t*-test was used to assess significant differences.

A good correlation was observed between biological replicates (Figure 3). Sixteen miRNAs were significantly differentially expressed between the *G. intraradices* inoculation and non-inoculation roots. Of those, zma-miR397b-5p, zma-miR528b-3p, and 12 miRNAs belonging to the miR399 family were significantly down-regulated, while zma-miR167g-3p and zma-miR159d-3p were significantly up-regulated (Figure 3, Table S6). To confirm the expression of AM fungus responsive miRNAs, nine of them were verified by quantitative reverse transcription polymerase chain reaction (RT-qPCR). A similar expression pattern was observed in results between RT-qPCR and sequencing data (Figure 3, Figure 4 and Figure S2), indicating that the relative expression level identified by the sequencing data was reliable and suitable for further analyses. Twelve of the DEMs were members of the miR399 family, including zma-miR399d-5p, zma-miR399h-3p, zma-miR399b-3p, zma-miR399a-5p, zma-miR399g-3p, zma-miR399f-3p, zma-miR399b-5p, zma-miR399j-5p, zma-miR399j-3p, zma-miR399d-3p, zma-miR399h-5p and zma-miR399f-5p.

### 2.5. Prediction of the Differentially Expressed miRNAs Target Genes

Prediction of DEMs targeting genes provided a way to understand the regulatory functions in symbiosis. Target gene prediction analysis shows that one miRNA targets multiple mRNAs while multiple miRNAs can target the same mRNA (Tables S7 and S8). Zma-miR167g-3p, zma-miR399h-5p, zma-miR399f-3p, and zma-miR528b-3p had the most targets, with 74, 67, 65, and 55 genes, respectively (Table S8). The predicted function of the target genes can be seen in Table S7.

### 2.6. Function and Enrichment Analyses of the Target Genes

**Figure 5:**
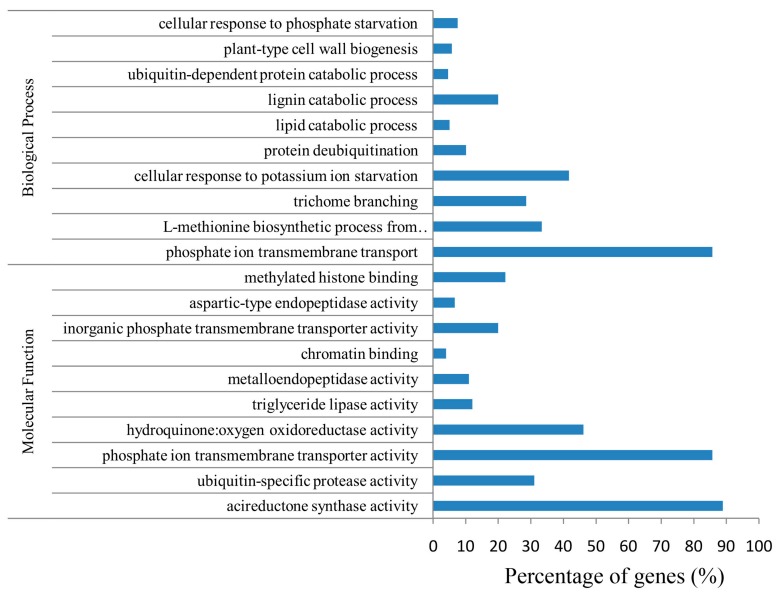
Gene Ontology (GO) term enrichments of the target genes of differentially expressed miRNAs (DEMs). The axis X represents the percentage of target genes to all genes in this category.

The Gene Ontology (GO) terms of the target genes were enriched into many enzymatic and transportation activities in the molecular function category, including acireductone synthase activity, phosphate ion transmembrane transporter activity, hydroquinone:oxygen oxidoreductase activity, ubiquitin-specific protease activity, methylated histone binding, inorganic phosphate transmembrane transporter activity, triglyceride lipase activity metalloendopeptidase activity, aspartic-type endopeptidase activity (Figure 5). The GO terms were enriched into starvation, transportation, and salvage processes in the biological process category, including cellular response to phosphate starvation, response to potassium ion starvation, phosphate ion transmembrane transport, and L-methionine biosynthetic process from methylthioadenosine trichome branching etc. (Figure 5). No GO term was enriched in the cellular component category.

**Figure 6:**
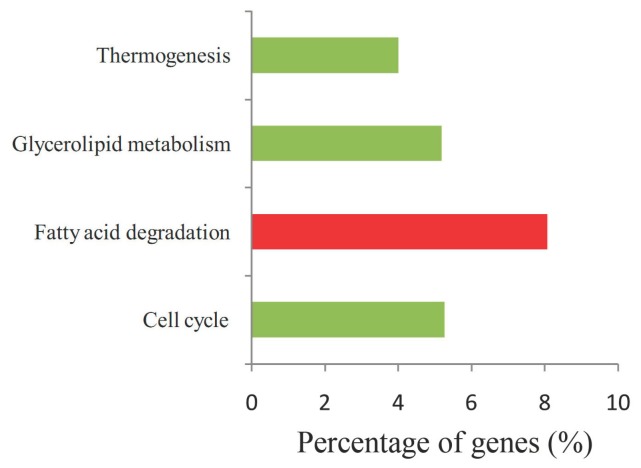
KEGG (Kyoto Encyclopedia of Genes and Genomes) pathway enrichment of the target genes of DEMs. The axis X the percentage of enriched target genes to all genes in this pathway. The red bar represents the pathway that was controlled by down-regulated miRNAs.

**Figure 7:**
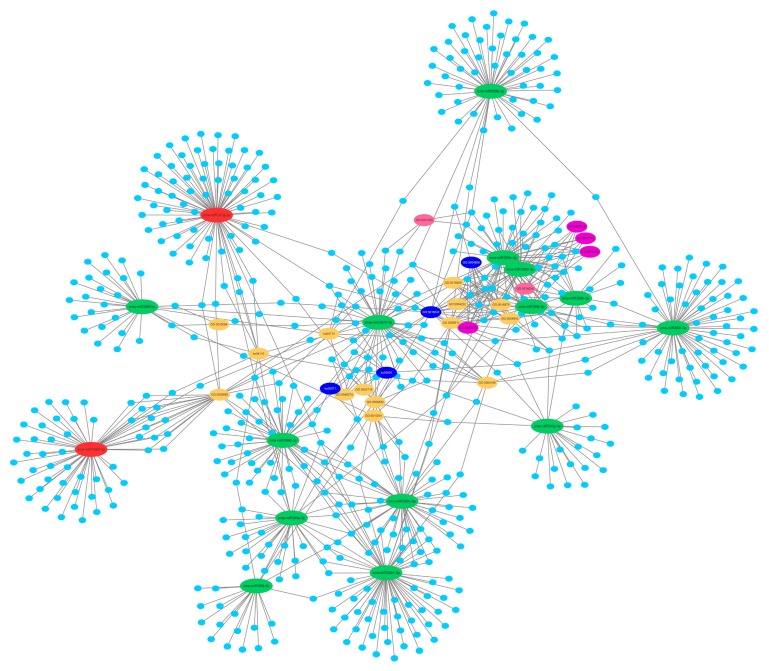
The potential regulating network of arbuscular mycorrhiza (AM)-responsive miRNAs in maize root. Red ovals represent up-regulated miRNAs; green ovals represent down-regulated miRNAs; blue ovals represent lip metabolism related pathways; purple ovals represent phosphate uptake related pathways; pink ovals represent starve responsive related pathways; yellow ovals represent the rest enriched pathways; and cyan circles represent the miRNA target genes.

KEGG (Kyoto Encyclopedia of Genes and Genomes) pathway analysis showed that the target genes were participated in glycerolipid metabolism, cell cycle, fatty acid degradation, and ubiquitin mediated proteolysis (Figure 6). Among them, the fatty acid degradation pathways containing most genes might play important roles in regulating maize root and AM symbiosis. The most complicated network consisting of 16 DEMs and 588 target genes were involved in regulating AM colonization (Figure 7, Table S7). In many cases, one target is shared by two or more miRNAs. For example, Zm00001d003797 (a root r-b1-like gene) was regulated by zma-miR399d-5p, zma-miR399b-5p, and zma-miR399h-5p. Except for zma-miR159d-3p and zma-miR167g-3p, the rest of the miRNAs in the network were down-regulated in response to AM fungus colonization. According to GO and KEGG enrichment analyses of miRNAs target genes in this network, it can be seen that the network was mainly involved in lipid metabolism, response to phosphate starvation, and the production and uptake of phosphate (Figure 7). The genes involved in fatty acid metabolism should be regulated by zma-miR399b-5p, zma-miR399h-5p and zma-miR397b-5p (Figure 7, Table S7).

## 3. Discussion

Plant–fungi symbiosis is an interesting research topic for its biological significance and applied potential. Many miRNA studies in plant have been reported but the miRNA profile in roots symbiosis is not well-explored. The symbiosis of AM fungi in maize root is an especially interesting interaction between roots in non-legume plant maize, a major crop, and fungi, which may improve maize plantation. Here, we reported 155 known and 28 novel miRNAs involved in maize root symbiosis with AM fungi via high-throughput sequencing. The novel miRNAs enrich our resources in maize and a new scenario of symbiosis at miRNA level. Our prediction of the miRNA targeting genes revealed that the crucial regulatory network of gene expression in maize during symbiosis, especially the lipid metabolism. Therefore, our results provide new insights to the roles of miRNAs in root–fungal symbiosis.

The 24-nt miRNAs are the major class of miRNAs, which is similar to previous observation in tomato and *N. tabacum* [1,24]. This indicates that different species might have undergone similar evolution to expand genes coding for 24-nt miRNAs. The miRNA processing pathways are different between 24 nt and those shorter than 24 nt miRNAs. Previous studies showed that distinct size miRNAs are generated by different DCL family members, i.e., 21 nt by *DCL1* and *DCL4*, 22 nt by *DCL2*, and 24 nt by *DCL3* [3]. It has been known that *OsDCL3a* is responsible for the biogenesis of 24-nucleotide long miRNAs in rice [25]. However, whether maize underwent the same evolutionary in producing 24-nt miRNA cannot be ascertained in this study.

AM fungi can form mutualistic symbiosis with approximately 80% of vascular plants in terrestrial ecosystems, delivering Pi to the host plant to enhance host Pi uptake [11]. Phosphate starvation response factor (PHR1) existing in many plants plays a central role in signaling of primary Pi responses [26,27]. PHR1 binds to P1BS elements (GNATATNC) and induces the expression of miRNAs members in the miR399 family [26]. Then members of miR399 can bind *PHO2* (PHOSPHATE2; ubiquitin-conjugating enzyme E2), resulting in degradation or translational repression of PHO2 [6]. PHO2 negatively regulates the expression of several Pi starvation inducible genes [6]. Thus, members of miR399 can suppress *PHO2* expression to promote the absorption of Pi uptake after a series of reaction. Besides, a recent investigation reported a potential regulatory role of miR399 in the establishment of the AM symbiosis in *M. truncatula*. In our study, zma-miR399d-5p, zma-miR399h-3p, zma-miR399b-3p, zma-miR399a-5p, zma-miR399g-3p, zma-miR399f-3p, zma-miR399b-5p, zma-miR399j-5p, zma-miR399j-3p, zma-miR399d-3p, zma-miR399h-5p and zma-miR399f-5p were down-regulated. This result is similar to that of miR399 in *M. truncatula*; however, more members of miR399 were significantly down-regulated by AM fungi colonization in maize than in *M. truncatula*. In most cases, the AM symbiosis resulted in decreased miR399 levels, and AM fungi could improve local Pi availability under low-Pi environments [17]. On the other hand, in response to the AM symbiosis, decreased levels of miR399 are able to increase *PHO2* expression, which is followed by suppressing Pi starvation-inducible genes in the direct Pi-uptake pathway, leading to a reorganization of Pi uptake. Gu et al. (2010) also observed that the miR399 family members *sly-miR399h* and *sly-miR399m* in the tomato root were responsive to AM colonization [28]. The down-regulation pattern of zma-miR399 of monocot was similar to that in dicot *M. truncatula* and tomato, which suggests that miR399 was a conserved miRNA family to be involved in AM symbiosis in different plants.

After establishment of symbiosis relationship, plants will absorb Pi from remote soil through AM fungal mycelia while AM fungi will acquire carbohydrates from their hosting plant [11]. A recent breakthrough found that AM fungi receiving the major source of organic carbons delivered to the fungus are lipids but not in the form of sugars [29,30]. However, how the lipid metabolic pathway is regulated during the symbiosis is unclear. Results of our study showed that lipid metabolism might be regulated by differentially expressed miRNAs via their target genes. Zma-miR399b-5p, zma-miR399h-5p and zma-miR397b-5p should control the expression of genes for fatty acid metabolism and/or transportation. The decreased expression of the three miRNAs resulted in an increase of fatty acid biosynthetic process genes expression, which promotes fatty acid metabolism and delivering to AM fungi to maintain symbiosis status. In human, miR-122 is a key regulator of cholesterol and fatty acid metabolism in the adult liver [31], while miR-33a/b is the regulator of fatty acid metabolism and insulin signaling [32]. Apparently, the key miRNAs that control the fatty acid metabolism should be different between plants and human.

MiR167g is significantly up-regulated after AM fungi inoculation in our study. Similarly, miR167c in soybean (*Glycine max*) roots were also up-regulated after inoculation with *Bradyrhizobium japonicum* strain USDA110 (the microsymbiont) [33]. In soybean, miR167 acts as a positive regulator of lateral root and nodulation development by repressing the target genes *GmARF8a* and *GmARF8b* (ARF, Auxin response factors) [33]. Therefore, the up-regulation of miR167g in *G. intraradices* -infected maize root indicated that it might play important roles in regulating and maintaining the status of AM-symbiosis. MiR167 was also reported in response to salt stress and water stress by regulating target genes in maize and *Arabidopsis*, respectively [23,34], suggesting that miR167 can also be involved in the regulation of resistance to abiotic stress under symbiotic conditions.

The production and uptake of phosphate were also the important targets of DEMs. Many members of the miR399 family might control the expression of phosphate starvation responsive, transport and homeostasis genes, which play roles in symbiotic Pi transport and Pi signaling in symbiosis. These miRNAs in this network were down-regulated in response to symbiosis, which resulted in the up-regulation of corresponded phosphate transport-related gene expression, Pi transportation from AM fungi to plants, and further mycorrhizal formation. Similar results were also observed in many studies [6,17,28]. Together, it is very interesting that several members in miR399 family and miR397 family might control fatty acid metabolism and promote delivering of lipids from plants to AM fungi. All the facts indicate that the exchange process of lipids and phosphate between plant and AM fungi should be coordinately regulated by miRNAs.

## 4. Materials and Methods

### 4.1. Plant Materials

Maize (*Zea mays*) B73 kernels were surface sterilized by 15% bleach for 15 min at room temperature, and then the seeds were washed with ddH_2_O for three times. A small sterilized sieve (20 cm in diameter) was used to drain out water after each wash. After washing, kernels were kept on a double-faced brown filter paper and left for 10 min until the seeds were dry. Seeds were germinated on 0.8% water agar plates at 28 °C for 7 days. After germinating, three seedlings were transferred to a pot containing 1:3 ceramists:sand mixture. Treated roots were inoculated with *G. intraradices* while the control roots were added the same amount of autoclaved inoculum. Plants were cultivated under 16 h light and 8 h dark at 28 °C. All plants were watered one time per week with a modified Hoagland solution containing 50 µM phosphates [11]. The roots of maize seedlings at 40 days old after inoculation were harvested for subsequent analyses. Three repeats were performed. Three plants were used for each repeat and each treatment.

### 4.2. Detection of Mycorrhizal Colonization

First, maize roots were washed and fixed in FAA (formalin–acetic acid–alcohol) for 4 h. Roots were then treated with 10% KOH and heated for 1 h, followed by 2% HCl solution treatment for 5 min. Finally, roots were stained with Trypan Blue [11]. Dyed roots were detected after transparency at lactic acid and glycerin.

### 4.3. RNA Extraction, Small RNA Library Preparation and Sequencing

Total RNA was isolated from maize roots using RNiso Plus reagent (Takara, Dalian, China), and then was treated with RNAase-free DNAase I according to the manufacturer’s instructions. The integrity of RNA was examined on an Agilent 2100. The RNA was run on 15% PAGE (polyacrylamide gel), and then small RNAs (sRNAs) fractions of 18–30 nt were separated, extracted and eluted. A pair of adaptors was ligated to the 5’ and 3’ ends of the sRNAs followed by quantitative reverse transcription PCR [35]. The PCR products were checked for quality and quantified via a Bioanalyzer (Agilent, Waldbronn, Germany). The purified PCR products were used for sequencing analysis by BGISEQ-500 (BGI, Shenzhen, China). Six libraries were sequenced in this study.

### 4.4. Identification of Known miRNAs and Novel miRNAs

Raw data were cleaned to remove low-quality and adaptor sequences. Then, the clean reads (SRA accession: SRP134940) were mapped onto the maize B73 genome (RefGen_V4 genome) with BWA under default parameters [36,37]. After that, perfectly matched sequences were compared with maize miRNAs in the miRBase 22 database (Accessed online: http://www.mirbase.org/) to identify the known miRNAs. The reads matched to rRNA, tRNA, snRNA, snoRNA and other non-coding RNA sequences deposited in Rfam 11.0 database and the repeat-Repbase (Accessed online: http://www.girinst.org/repbase) were deducted. ShortStack (version 3.8.5) was used to identify novel miRNAs [38]. The novel miRNAs were classified and nominated according to Meyers et al. (2008) [39]. The precise position of novel miRNAs on the maize genome v4 was verified by software BLAT [40] and manually corrected.

### 4.5. Identification of AM Fungus Responsive miRNAs

To identify AM symbiosis-responsive miRNAs, raw miRNA counts from each sample were used to normalize expressions of miRNAs. The fold changes of miRNAs levels in treated samples were calculated relative to that in control. The miRNAs with fold-change in expression log_2_(treatment/control) ≥ 1 and adjusted *p* value < 0.05 were considered as differentially expressed miRNAs, and referred to as the AM fungus-responsive miRNAs. Tool DEseq (version 1) was used to identify the differentially expressed miRNAs [41].

### 4.6. Stem-Loop Reverse Transcription Polymerase Chain Reaction (RT-qPCR)

Stem-loop specific reverse transcription was performed according to Chen et al. (2005) [42]. Reverse transcriptase reactions contained DNase-treated total RNA and a gene-specific stem-loop RT primer. MiRNA PrimeScript RT Enzyme Mix Kit was used for reverse transcriptase reactions according to the manual. After transcription, cDNA samples were used as templates for RT-qPCR analysis. The RT-qPCR was performed by SYBR^®^ PrimeScript™ miRNA RT-PCR Kit (RR716, Takara, Dalian, China) according to the instruction. The *5S rRNA* was used as a reference gene and normalization control [19]. All primers used for stem-loop RT-qPCR are listed in Table S9. The relative expression level of the miRNAs was calculated by the 2^−ΔΔCt^ method. Three biological replicates with three technical replicates were performed for each sample.

### 4.7. Identification of miRNA Targets

The putative target genes were predicted by the psRNATarget algorithm (Accessed online: http://plantgrn.noble.org/psRNATarget). The prediction rules were as follows: (1) maximum expectation is less than 5.0; (2) length for complementary scoring (hspsize) is shorter than 19; (3) range of central mismatch leading to translational inhibition is 10–11 nt. The mature sequences of all miRNAs were submitted to predict target genes against the library (all the *Zea mays* transcripts, version 4.0) [37]. To elucidate the regulatory function and pathways involved in symbiosis, the predicted target genes were further annotated against GO and enriched by using Blast2GO (version 2.8, Accessed online: http://www.blast2go.com), and against the KEGG pathway database using KAAS (KEGG Automatic Annotation Server, Accessed online: https://www.genome.jp/kegg/kaas/), respectively. The relationship pairs between miRNA and target gene, between target gene and GO terms, between target gene and the KEGG pathway were used as the edges during network construction. The miRNA, target gene and GO terms and KEGG pathway were used as the nodes. The network was visualized via Cytoscape (version 3.6.0) [43].

## 5. Conclusions

A total of 155 known maize miRNAs and 28 novel miRNAs were identified. Fourteen miRNAs were significantly down-regulated, and two were up-regulated in maize root responsive to *G. intraradices* inoculation. Of the known DEMs, 12 down-regulated miRNAs belonged to the miR399 family. The differentially expressed miRNAs might regulate lipid metabolism, response to phosphate starvation, and the production and uptake of phosphate of the plant during the symbiosis processes via the target genes.

## Supplementary Materials

Supplementary materials can be found at http://www.mdpi.com/1422-0067/19/10/3201/s1.
