# Supplementary material for: Identification of Arbuscular Mycorrhiza Fungi Responsive microRNAs and Their Regulatory Network in Maize
Source: Int J Mol Sci. 2018 Oct 16;19(10):3201. doi: 10.3390/ijms19103201 (PMC6214007; doi:10.3390/ijms19103201)
Supplement: Supplementary file 1 [file ijms-19-03201-s001.zip › Table S1.docx]

Table S1 Statistics of sequencing reads for six libraries from maize

| Name | Total reads | Mapping reads |
| --- | --- | --- |
| Control-1 | 1409445 | 1263406 (89.64%) |
| Control-2 | 1100181 | 993373 (90.29%) |
| Control-3 | 1873234 | 1681122 (89.74%) |
| Treatment -1 | 1570135 | 1297742 (82.65%) |
| Treatment -2 | 836018 | 698068 (83.50%) |
| Treatment -3 | 1590230 | 1349677 (84.87%) |
