# Supplementary material for: Identification of Arbuscular Mycorrhiza Fungi Responsive microRNAs and Their Regulatory Network in Maize
Source: Int J Mol Sci. 2018 Oct 16;19(10):3201. doi: 10.3390/ijms19103201 (PMC6214007; doi:10.3390/ijms19103201)
Supplement: Supplementary file 1 [file ijms-19-03201-s001.zip › Table S2.docx]

Table S2 The number of reads distribution of short RNA sequences

| Length (nt) | Treatment reads | Control reads |
| --- | --- | --- |
| 18 | 1419925.00 | 850515.00 |
| 19 | 1844920.00 | 1233296.00 |
| 20 | 1707602.33 | 1380840.33 |
| 21 | 2991555.67 | 2717960.67 |
| 22 | 2396917.67 | 2193604.33 |
| 23 | 1975111.33 | 1960422.67 |
| 24 | 5716702.67 | 6803319.00 |
| 25 | 1160798 | 944736.6667 |
| 26 | 847053.3333 | 685735 |
| 27 | 700749.3333 | 539504.6667 |
| 28 | 386878.3333 | 254306.6667 |
