# Supplementary material for: Identification of Arbuscular Mycorrhiza Fungi Responsive microRNAs and Their Regulatory Network in Maize
Source: Int J Mol Sci. 2018 Oct 16;19(10):3201. doi: 10.3390/ijms19103201 (PMC6214007; doi:10.3390/ijms19103201)
Supplement: Supplementary file 1 [file ijms-19-03201-s001.zip › Table S3.docx]

Table S3 Numbers of different types of other non-coding RNAs in two libraries

| RNA | Treatment | Control |
| --- | --- | --- |
| rRNA | 2125 | 1962 |
| snoRNA | 1762 | 1622 |
| otherNcRNA | 12917 | 12142 |
| tRNA | 222 | 195 |
| snRNA | 4336 | 4981 |
| unAnnotation | 1281748 | 1086173 |
