# Supplementary material for: Identification of Arbuscular Mycorrhiza Fungi Responsive microRNAs and Their Regulatory Network in Maize
Source: Int J Mol Sci. 2018 Oct 16;19(10):3201. doi: 10.3390/ijms19103201 (PMC6214007; doi:10.3390/ijms19103201)
Supplement: Supplementary file 1 [file ijms-19-03201-s001.zip › Table S4.docx]

Table S4 Family information of known miRNAs identified from maize root

| miRNA family | miRNA name | miRNA family | miRNA name |
| --- | --- | --- | --- |
| zm-miR156 | zma-miR156a-3p | zm-miR171 | zma-miR171a-5p |
|  | zma-miR156b-3p |  | zma-miR171e-5p |
|  | zma-miR156e-3p |  | zma-miR171f-3p |
|  | zma-miR156g-3p |  | zma-miR171f-5p |
|  | zma-miR156h-3p |  | zma-miR171g-3p |
|  | zma-miR156i-3p |  | zma-miR171g-5p |
|  | zma-miR156j-5p |  | zma-miR171h-5p |
|  | zma-miR156k-3p |  | zma-miR171i-3p |
|  | zma-miR156k-5p |  | zma-miR171i-5p |
|  | zma-miR156l-3p |  | zma-miR171j-5p |
|  | zma-miR156l-5p |  | zma-miR171l-5p |
| zm-miR159 | zma-miR159a-5p |  | zma-miR171m-3p |
|  | zma-miR159d-3p |  | zma-miR171m-5p |
|  | zma-miR159d-5p |  | zma-miR171n-3p |
|  | zma-miR159e-3p |  | zma-miR171n-5p |
|  | zma-miR159f-5p | zm-miR172 | zma-miR172b-5p |
|  | zma-miR159i-3p |  | zma-miR172c-3p |
|  | zma-miR159k-3p |  | zma-miR172c-5p |
| zm-miR160 | zma-miR160a-3p |  | zma-miR172e |
|  | zma-miR160d-3p | zm-miR2118 | zma-miR2118b |
|  | zma-miR160f-3p |  | zma-miR2118c |
|  | zma-miR160f-5p |  | zma-miR2118d |
|  | zma-miR160g-3p |  | zma-miR2118g |
| zm-miR162 | zma-miR162-3p | zm-miR2275 | zma-miR2275c-5p |
|  | zma-miR162-5p |  | zma-miR2275d-5p |
| zm-miR164 | zma-miR164a-3p | zm-miR319 | zma-miR319c-5p |
|  | zma-miR164b-3p |  | zma-miR319d-3p |
|  | zma-miR164d-3p |  | zma-miR319d-5p |
|  | zma-miR164e-3p | zm-miR390 | zma-miR390b-3p |
|  | zma-miR164e-5p |  | zma-miR390b-5p |
|  | zma-miR164f-3p | zm-miR393 | zma-miR393a-3p |
|  | zma-miR164f-5p |  | zma-miR393b-3p |
|  | zma-miR164g-3p |  | zma-miR393c-3p |
|  | zma-miR164g-5p |  | zma-miR393c-5p |
|  | zma-miR164h-3p | zm-miR394 | zma-miR394b-3p |
|  | zma-miR164h-5p |  | zma-miR394b-5p |
| zm-miR166 | zma-miR166a-3p | zm-miR395 | zma-miR395a-5p |
|  | zma-miR166a-5p |  | zma-miR395o-3p |
|  | zma-miR166c-5p |  | zma-miR395p-5p |
|  | zma-miR166d-3p | zm-miR396 | zma-miR396a-3p |
|  | zma-miR166d-5p |  | zma-miR396a-5p |
|  | zma-miR166g-5p |  | zma-miR396d |
|  | zma-miR166h-5p |  | zma-miR396f-3p |
|  | zma-miR166i-5p |  | zma-miR396f-5p |
|  | zma-miR166l-5p |  | zma-miR396g-3p |
|  | zma-miR166m-3p |  | zma-miR396h |
|  | zma-miR166m-5p | zm-miR397 | zma-miR397b-3p |
|  | zma-miR166n-3p |  | zma-miR397b-5p |
| zm-miR167 | zma-miR167a-3p | zm-miR398 | zma-miR398a-5p |
|  | zma-miR167b-3p |  | zma-miR398b-3p |
|  | zma-miR167c-3p |  | zma-miR398b-5p |
|  | zma-miR167c-5p | zm-miR399 | zma-miR399a-5p |
|  | zma-miR167d-3p |  | zma-miR399b-3p |
|  | zma-miR167e-3p |  | zma-miR399b-5p |
|  | zma-miR167f-3p |  | zma-miR399c-5p |
|  | zma-miR167g-3p |  | zma-miR399d-3p |
|  | zma-miR167i-3p |  | zma-miR399d-5p |
|  | zma-miR167j-3p |  | zma-miR399e-5p |
|  | zma-miR167j-5p |  | zma-miR399f-3p |
| zm-miR168 | zma-miR168a-3p |  | zma-miR399f-5p |
|  | zma-miR168b-3p |  | zma-miR399g-3p |
|  | zma-miR168b-5p |  | zma-miR399h-3p |
| zm-miR169 | zma-miR169b-3p |  | zma-miR399h-5p |
|  | zma-miR169b-5p |  | zma-miR399i-5p |
|  | zma-miR169c-3p |  | zma-miR399j-3p |
|  | zma-miR169e |  | zma-miR399j-5p |
|  | zma-miR169f-3p | zm-miR408 | zma-miR408b-3p |
|  | zma-miR169h |  | zma-miR408b-5p |
|  | zma-miR169j-3p | zm-miR444 | zma-miR444b |
|  | zma-miR169j-5p | zm-miR528 | zma-miR528b-3p |
|  | zma-miR169l-5p |  | zma-miR528b-5p |
|  | zma-miR169m-3p | zm-miR529 | zma-miR529-5p |
|  | zma-miR169p-5p | zm-miR827 | zma-miR827-3p |
|  | zma-miR169q-5p |  | zma-miR827-5p |
|  | zma-miR169r-3p | zm-miR11969 | zma-miR11969-5p |
|  | zma-miR169r-5p | zm-miR11970 | zma-miR11970-3p |
| zm-miR1432 | zma-miR1432-3p |  | zma-miR11970-5p |
|  | zma-miR1432-5p |  |  |
