# Supplementary material for: Identification of Arbuscular Mycorrhiza Fungi Responsive microRNAs and Their Regulatory Network in Maize
Source: Int J Mol Sci. 2018 Oct 16;19(10):3201. doi: 10.3390/ijms19103201 (PMC6214007; doi:10.3390/ijms19103201)
Supplement: Supplementary file 1 [file ijms-19-03201-s001.zip › Table S8.docx]

Table S8 The number of target genes of each differentially expressed miRNA

| miRNA | Number of target genes |
| --- | --- |
| zma-miR167g-3p | 74 |
| zma-miR399h-5p | 67 |
| zma-miR399f-3p | 65 |
| zma-miR528b-3p | 55 |
| zma-miR399b-3p | 54 |
| zma-miR159d-3p | 54 |
| zma-miR399b-5p | 53 |
| zma-miR399d-5p | 47 |
| zma-miR397b-5p | 47 |
| zma-miR399a-5p | 34 |
| zma-miR399d-3p | 32 |
| zma-miR399f-5p | 29 |
| zma-miR399g-3p | 25 |
| zma-miR399j-3p | 21 |
| zma-miR399j-5p | 20 |
| zma-miR399h-3p | 15 |
