# Supplementary material for: Identification of Arbuscular Mycorrhiza Fungi Responsive microRNAs and Their Regulatory Network in Maize
Source: Int J Mol Sci. 2018 Oct 16;19(10):3201. doi: 10.3390/ijms19103201 (PMC6214007; doi:10.3390/ijms19103201)
Supplement: Supplementary file 1 [file ijms-19-03201-s001.zip › Table S9.docx]

Table S9 Primers used in this study

| Names | Primers |
| --- | --- |
| RTmiR399a-5p | GTGCGGTTCTCCTCTGGCACG |
| RTmiR399b-5p | GTGCAGCTCTCCTCTGGCATG |
| RTmiR399h-3p | TGCCAAAGGAGAATTGCCCTG |
| RTmiR399f-5p | GGGCAACTTCTCCTTTGGCAGA |
| RTmiR399g-3p | TGCCAAAGGGGATTTGCCCGG |
| RTmiR399h-5p | GTGCAGTTCTCCTCTGGCACG |
| RTmiR528b-3p | CCTGTGCCTGCCTCTTCCATT |
| RTmiR399b-3p | TGCCAAAGGAGAGCTGTCCTG |
| RTmiR167g-3p | GGTCATGCTGTAGTTTCATC |
| RT-5S | GATCCCATTCCGACCTCGATATA |
