# Supplementary material for: Identification of Arbuscular Mycorrhiza Fungi Responsive microRNAs and Their Regulatory Network in Maize
Source: Int J Mol Sci. 2018 Oct 16;19(10):3201. doi: 10.3390/ijms19103201 (PMC6214007; doi:10.3390/ijms19103201)
Supplement: Supplementary file 1 [file ijms-19-03201-s001.zip › Supplementary figures.doc]

Figure S1 Trypan blue staining of maize root colonized by *Rhizophagus irregularis*

Figure S2 Sequencing expression data of miRNAs in Figure 4
